# The Effects of Nasal Septal Deviation on Anterior Skull Base Parameters

**DOI:** 10.1055/s-0043-1773760

**Published:** 2024-03-11

**Authors:** Şeyda Akbal Çufalı, Mehmet Ali Çetin, Nurcan Yurtsever Kum, Süleyman Emre Karakurt

**Affiliations:** 1 Department of Otolaryngology, Ankara City Hospital, Cankaya, Turkey; 2 Department of Otolaryngology, İzmir Tepecik Training and Research Hospital, İzmir, Turkey

**Keywords:** skull base, nasal septum, olfactory groove, cribriform plate and horizontal plate of ethmoid bone

## Abstract

**Introduction** Functional endoscopic sinus surgery and endoscopic skull base surgery are frequently performed surgeries today. Nasal septal deviation is a common finding and can affect the surgical area. Therefore, it is important to examine the effect of this deviation on other anatomical structures.

**Objectıve** The aim of the present study was to determine whether there is a relationship between the degree of nasal septal deviation and anterior skull base structures using computed tomography (CT).

**Methods** A total of 312 patients (aged 18 to 65 years old) whose paranasal sinus CT images were available were included in the study. Measurements were obtained on images retrieved from Picture Archiving and Communication System (PACS) and Horos image archive systems in the bone window in the coronal and axial plane.

**Results** The mean age of 312 patients was 33.00 years old (standard deviation [SD] 11.22 years). The presence of septal deviation was not associated with changes in olfactory fossa (OF) depths, Keros degrees, and the angle between the lateral lamella and the cribriform lamella. However, OF depths and Keros degrees on the deviated side of the septum were found to change at a significant level ( *p* < 0.05). No significant association was observed between the degree of septal deviation and cribriform lamella-lateral lamella angle.

**Conclusion** The study showed significantly increased OF depth and Keros degree on the deviated side of the nasal septum. Performing CT scans before endoscopic sinus surgery and endoscopic skull base surgery is important to increase the chances of a successful surgical outcome and to reduce complications.

## Introduction

The nasal septum is one of the key structures in nasal surgery. The septum helps regulate smooth airflow through the nasal cavities. Its main functions are to provide support and stability to the external nose and to form a foundation for the nasal mucosa. Nasal septum deviation is among the most common causes of nasal obstruction. 1 2

Iatrogenic traumas have been increasingly recognized as a result of widespread availability of endoscopic sinus surgery due to technological advances and a better understanding of the paranasal sinuses. Cerebrospinal fluid (CSF) leak is the most prevalent event following trauma. At the junction of the thicker orbital rim of the frontal bone and the thin lateral lamina of the cribriform plate, there is a connection that extends to the posterior part of the recessus frontalis. This connection is in close relation with the anterior skull base. This is the area where CSF leak occurs most frequently due to penetration during surgery. 2

The Keros classification is based on the length of the lateral lamella that joins the cribriform plate to the fovea ethmoidalis of the frontal bone and is used as an important parameter in predicting the depth of the skull base in endoscopic sinus surgery 3 4

High-resolution computed tomography (CT) (also known as thin-section CT scanning) is particularly useful for anatomical evaluation of the nasal septum and skull base to prevent complications and residual/recurrent diseases. Measurements are obtained from the bone windows. 5 A variety of methods have been reported in the literature to measure nasal septum deviation. The present study aimed to obtain data that could be used to improve the safety of endoscopic sinus surgery by comparing the septal deviation angle with the key parameters for anterior skull base surgery using CT.

## Materials and Methods

The present study was initiated after obtaining approval from the local ethics committee (Approval No: E-19–2608). A total of 350 patients (age range, 18 to 65 years old) who presented to the otorhinolaryngology outpatient clinic of Ankara Numune Research and Training Hospital with complaint of nasal obstruction and underwent paranasal sinus CT imaging since January 2014 were included in the study. The study had a retrospective design. Patients with a history of previous trauma or surgery on the nose, and patients with a sinonasal tumor, sinonasal polyposis, acute rhinosinusitis, CSF leak and significant facial deformity were excluded from the study.

**Fig. 1:**
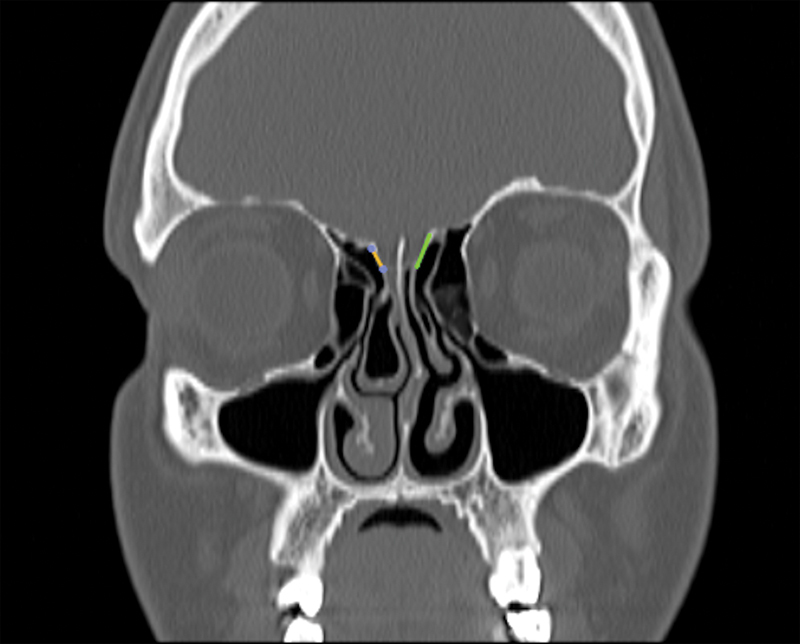
Lateral lamella measurement technique

**Fig. 2:**
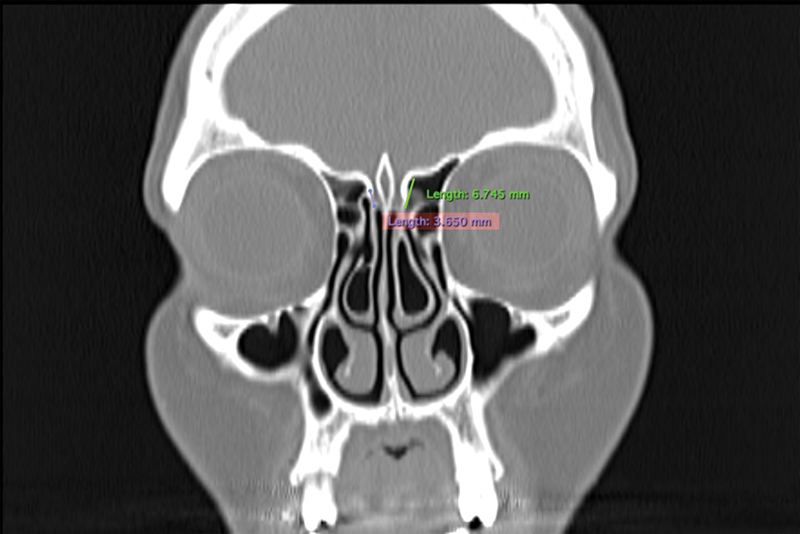
Lateral lamella measurement

**Fig. 3:**
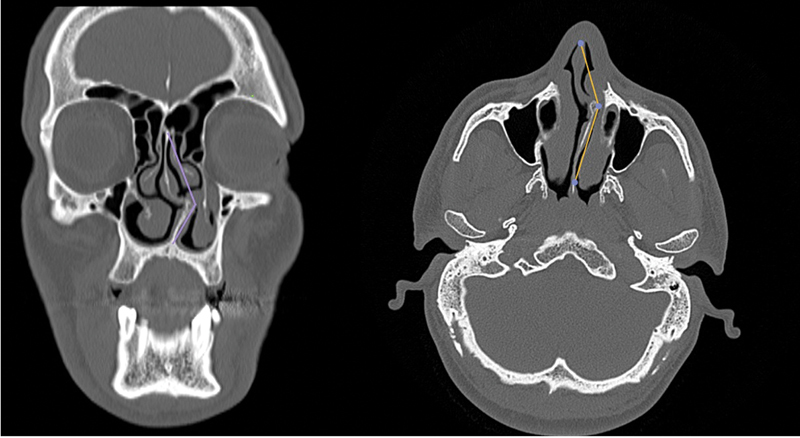
Measurement of septal deviation angle

Computed tomography imaging was performed using a multi-detector spiral CT scanner (Toshiba Alexion Advance 16, 2008 Japan). Computed tomography parameters were 120 KVP, 100–150 mA, minimum slice thickness of 0.5 mm (axial) and matrix size of 512 × 512. Images retrieved from Picture Archiving and Communication System (PACS) and Horos image archive systems were evaluated in the bone window with 1-mm sections in the axial and coronal plane. The infraorbital nerve point, the fovea ethmoidalis and medial ethmoid roof point, the lowest point of the lateral lamella of the cribriform plate, and the anterior ethmoidal artery point were determined as standard anatomic landmarks. Measurements were taken after marking these points ( Fig. 1 and Fig. 2 ). The angle of septal deviation was determined in the coronal plane by drawing a line from the nasal spine to the point where the septum was most deviated and another line to the base of the crista galli in the midline ( Fig. 3 ). In measurements made in axial sections, the distance between the midline passing through the anterior nasal spine and the posterior nasal spine and the section where the deviation is most severe was measured. The length of a vertical line drawn horizontally from the medial ethmoid roof was defined as the medial ethmoid roof height. The length of a vertical line drawn from the cribriform plate to the horizontal plane was defined as the height of the cribriform plate. The height of lateral lamella was estimated by subtracting the cribriform height from the medial ethmoid roof height. The depth of the olfactory fossa was categorized as Keros Type 1 (depth 1 to 3 mm), Keros Type 2 (depth 4 to 7 mm) and Keros Type 3 (depth 8 to 16 mm). Statistical analyses were based on these values. All measurements on CT images were performed by the same otorhinolaryngologists.

**Fig. 4:**
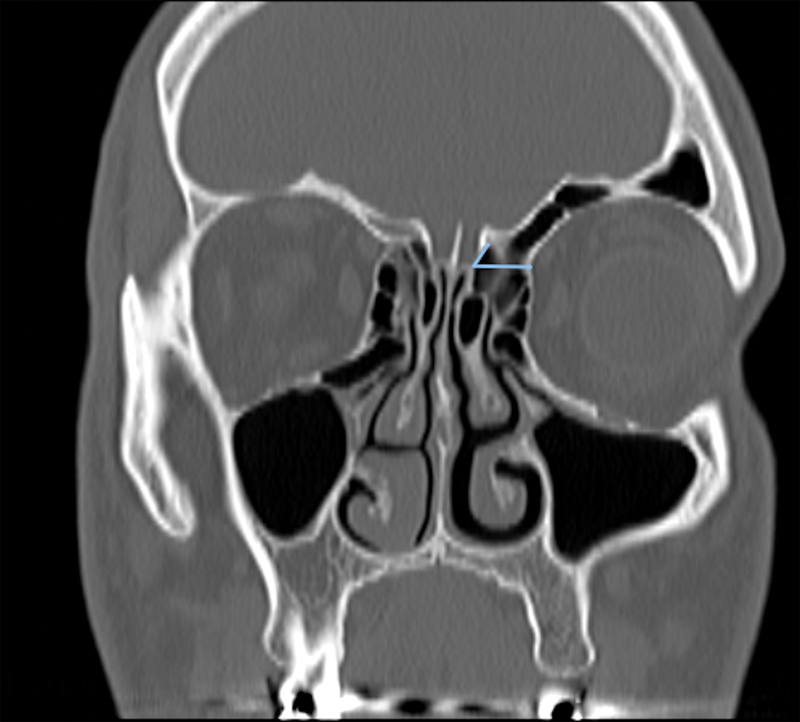
The angle between lateral lamella and cribriform lamella

The angle between the cribriform lamella and lateral lamella was determined by combining the plane running perpendicular to the orbit from the cribriform lamella and the axis of the lateral lamella ( Fig. 4 ). Except for the septal deviation angle, all measurements were obtained bilaterally.

## Statistical Analysis

The study parameters were analyzed using STATA 14.2 (StataCorp LP, College Station, TX, USA) in terms of statistical significance and associations. Age, sex, presence of a deviated septum, direction, and measured angles of septal deviation, right/left OF depths, right/left OF angles, and Keros types for the right and left sides were recorded for 312 patients and the effects of septal deviation angle on the skull structure was examined statistically based on these data. The statistical method used for the study is described below. Initially, the statistical features of the sample data were summarized and whether the nonparametric data distribution was close to normal was checked using the Kolmogorov-Smirnov and the Shapiro-Wilk tests as well as visualization of a histogram. The Pearson chi-squared test was used to analyze independence of categorical variables. Analysis of variance (ANOVA) was also used to test the independence of the groups. After confirming the suitability of the sample data for the study, the associations among dependent variables related to the anterior skull base structure and the degree of septal deviation using the Spearman correlation test both independently and depending on the direction of association. The significance levels were set at 5% for all statistical analyses ( *p* < 0.05).

## Results

At the beginning, 350 patients were recruited and, ultimately, 312 patients were included in the present study. However, information on age was available for 306 patients. The mean age ± SD of 306 patients was 33.00 ± 11.22 years. Septal deviation was not detected in 43 out of 312 patients. The mean age (±SD) was 34.95 ± 13.90 years old for patients without septal deviation and 32.68 ± 10.71 years old for patients with septal deviation ( Table 1 ).

**Table 1:** Associations among age, olfactory fossa depth, olfactory fossa angle and septal deviation

Variable	*n*	Mean	Std. Dev.	Min	Max
AGE	306	33.003	11.218	18	68
ROFDEPTH	312	5.753	2.154	1	13
RCPDEG	312	69.977	15.012	67	165
LOFDEPTH	312	5.512	1.994	1	12.3
LCPDEG	312	68,59	14.240	65.6	160
SDDEG coronal	312	144.455	21.323	92.8	180
SDDEG axial	312	146.75	13.2	120.3	180

Abbreviations: LCPDEG, Left Cribriform Plate Angle; LOFDEPTH, Left Olfactory Fossa Depth; RCPDEG, Right Cribriform Plate Angle; ROFDEPTH, Right Olfactory Fossa Depth; SDDEG, Septal Deviation Angle.

Out of the 312 patients included in the study, 105 were female and 207 were male. Septal deviation was absent in 86 out of 105 females and in 183 out of 207 males.

From the CT images reviewed, it was found that the mean depth was 5.75 ± 2.15 mm for the right OF and 5.51 ± 1.99 mm for the left OF. However, when the angle between the lateral lamella and cribriform lamella was examined, it was 69.9 ± 15° on the right side and 68.59 ± 21.3° on the left side. The mean septal deviation angle of the entire study population was 144.45 ± 21.32° in the coronal plane. The mean septal deviation angle of the entire study population was 146.75 ± 13.2°. High deviation angle was used in calculations. On the right side, the angle between the cribriform plate and the lateral lamella was measured a minimum of 67° and a maximum of 165°. The corresponding figures were a minimum of 65.6° and a maximum of 160° on the left side (shown in Table 1 ).

It was evaluated whether the parameters were normally distributed or not. At the significance ( *p* ) level of < 0.05, right/left OF depth and left OF angle values showed a normal distribution and the distribution of right OF angle values was close to normal. Values for age and septal deviation angles were not normally distributed. The anterior skull base parameters of the study sample followed a normal distribution ( *p* < 0.05) and therefore, deemed suitable for statistical analysis.

For the results of the normal distribution test among only patients with septal deviation, all variables except age were found to be normally distributed at a significance level of *p* < 0.05. Thus, the data were considered suitable for statistical analysis.

The independence of categorical variables (age, sex) and groups was analyzed. The results showed that septal deviation and direction of the septal deviation were independent of age and sex.

Statistically, both septal deviation and directions were independent of age and sex. The independence of anterior skull data from septal deviation showed independence of septal deviation direction at a significance level of *p* < 0.05. No statistically significant association was found between septal deviation and the right lateral lamella - cribriform lamella angle and the left lateral lamella - cribriform lamella angle ( *p* < 0.05).

From the table above showing septal deviation and anterior skull base parameters, it was observed that the presence of septal deviation did not change the depths of OFs, Keros degrees and the angle between the lateral lamella and the cribriform lamella ( *p* > 0.05).

The results presented above show that both categorical variables and other parameters are independently distributed and follow a normal distribution in the presence of septal deviation. The presence of a deviated septum was analyzed in both directions and for the right- and left-sided deviations separately. The Spearman correlation test was also used to determine whether the septal deviation angle was statistically correlated with the skull base variables ( Table 2 ).

**Table 2:** Independence test for septal deviation and anterior skull base parameters

	Variable|	Probe > F
ROFDEPTH	SD	0.923
LOFDEPTH	SD	0.773
RKEROSDEG	SD	0.907
LKEROSDEG	SD	0.789
ROFDEG	SD	0.616
LOFDEG	SD	0.901

Abbreviations: ROFDEPTH, Right Olfactory Fossa Depth ; LOFDEPTH, Left Olfactory Fossa Depth; RKEROSDEG Right Keros Angle , LLKEROSDEG Left Keros Angle ROFDEG, Right Olfactory Fossa Angle; LOFDEG Left Olfactory Fossa Angle.

Deviated regions of the nasal septum and their degrees were evaluated. Deviation in the bony septum was observed to accompany some changes in the anterior skull base structure. However, the deviation of the cartilage septum did not have a significant effect on the anterior skull base parameters. The study results demonstrate that a significant increase in septal deviation affects both left and right OF depths ( *p* = 0.0095 for the right side and *p* = 0.0445 for the left side). In addition, the Keros degree was significantly affected by the septal deviation degree such that the right Keros degree was affected when the septum was deviated to the right (and the left Keros degree is affected when the septum is deviated to the left.) P-values were as follows: *p* = 0.167 for the right Keros degree and *p* = 0.053 for the left Keros degree at right-sided deviation; and *p* = 0.04 for the right Keros degree and *p* = 0.042 for the left Keros degree at left-sided deviation. This effect was also apparent on the direction-independent analysis depending on the sample size. Despite that, it was concluded that septal deviation affects Keros degree in the same direction since the interaction of septal deviation with opposite Keros degree was rejected at *p* > 0.05 level. All are shown in Table 3 .

**Table 3:** Correlations of septal deviation angle with anterior skull base variables

	SDDEG	SSDEG (R)	SDDEG (L)
SDDEG	1.000	1.000	1.000
SDDEG P values	0.000	0.000	0.000
ROFDEPTH	- 0.2102*	- 0.2234*	- 0.1739*
ROFDEPTH P values	0.0005	0.0095	0.0445
LOFDEPTH	- 0.2017*	- 0.1941*	- 0.1970*
LOFDEPTH P values	0.0009	0.0247	0.0225
RKEROSDEG	- 0.2020*	- 0.2064*	- 0.1675
RKEROSDEG P values	0.0009	0.0167	0.0530
LKEROSDEG	- 0.1747*	- 0.1646	- 0.1756*
LKEROSDEG P values	0.004	0.0573	0.0424
RCPDEG	0.0596	- 0.0147	0.0104
RCPDEG P values	0.331	0.1921	0.9051
LCPDEG	- 0.0646	0.0486	- 0.0183
LCPDEG P values	0.291	0.2343	0.8336

Abbreviations: SDDEG, Septal Deviation Angle; ROFDEPTH, Right Olfactor Fossa Depth; LOFDEPTH, Left Olfactor Fossa Depth; RKEROSDEG, Right Keros Angle; LKEROS Deg, Left Keros Angle; RCPDEG, Right Cribriform Plate Angle; LCPDEG, Left Cribriform Plate Angle.

## Discussion

Besides being the keystone for the nasal roof, the nasal septum has a crucial role in the regulation of nasal airflow. Septal deviation is the most common nasal septum condition with a prevalence varying from 20 to 40%. 3 6 7 The perpendicular plate of ethmoid bone is one of the most important components of the bone nasal septum. The lamina cribrosa of the ethmoid bone (also known as the cribriform plate) is part of the inferior segment of the anterior cranial fossa. Due to its anatomic structure, the ethmoid bone is included in the skull base in different shapes and angles. 8 For this reason, the association of septum and ethmoid bone and their variations in the skull base structure have been increasingly investigated.

Several classifications have been introduced for the characterization of septal deviation but none of them has widespread use. 9 In the present study, the angle between the nasal spine and the most deviated part of the septum and the midline of the skull base was used, being measured with CT in the axial and coronal plane. There is a critical anatomic junction among the septum, the ethmoid bone, and the anterior skull base. Due to the embryological development of the ethmoid bone and the structures to which it is associated, CT evaluation performed in the preoperative period is especially important in showing bone deviation and related skull base effects. In endoscopic sinus surgery, interventions directed at this site may potentially result in complications such as CSF leak, ethmoidal artery injury, and damage to the olfactory region. Therefore, it is of paramount importance to have a better understanding of these structures preoperatively to prevent many complications. 10 With this in mind, the present study aimed to better understand the relations among these structures.

The angle of septal deviation and its relations with the paranasal sinuses and the skull base have been investigated in several studies. In a CT study by Skorek et al. 11 examining 120 patients, Keros types 1, 2, and 3 were identified in 9.2, 75.8 and 15% of the cases, respectively. However, they reported that, despite the presence of statistically significant correlations, patient age, sex, and measurement side cannot be regarded as being clearly associated with critical measurements. Similarly, in the present study, no significant sex- or-age related difference was found in septal deviation in the adult patient population.

In a study by Keros, Keros type 2 was found in %70.2 of the individuals and it was reported that it was the most common Keros type in the general population. The same study also found a lower Keros degree on the right side when compared with the left side. 12 In line with previous studies, Keros type 2 was the most common type in our study at a rate of 64.1%, followed by Keros type 1 at 21.4%, and Keros type 3 at 14.5%. The Keros classification was based on the longest segment of the lateral lamella and the mean OF depths were 5.75 ± 2.15 mm on the right side and 5.51 ± 1.99 mm on the left side. Arıkan et al. performed measurements on both coronal and sagittal planes using CT in 30 patients. May et al. obtained similar measurements using lateral sinus radiography. Both Arıkan et al. and May et al. observed an asymmetry between right and left Keros degrees at a statistically nonsignificant level. 8 9 In other studies investigating the asymmetry of the lateral lamella, Adeel et al. 11 reported a mean lateral lamella height of 5.5 ± 2.1 mm on the right side and of 5.2 ± 2 mm on the left side as well as asymmetry in 94.8% of the patients. Similar to our findings, Erdem et al. 13 found a mean cribriform plate depth of 6.1 ± 2.3 mm on the right side and of 6.1 ± 2.2 mm on the left side with no significant difference between the opposite sides. Although no statistically significant difference was found between the sides on the direction-based analysis of Keros degrees, surgeons should be aware of different heights of the cribriform plate between the sides and a declining level of the skull base prior to sinus surgery.

Several studies in the literature looked at how a deviated septum may affect other anterior skull base parameters. Among these, Saylısoy et al. 14 evaluated 99 patients using paranasal CT scanning in coronal sections to identify relationships between septal deviation angle and cribriform plate (CP) width and found a nonsignificant difference between ipsilateral and contralateral sides in the mean CP width when compared by septal deviation side (CP width 4.69 ± 1.36 mm at the ipsilateral side and 4.58 ± 1.51 mm at the nondeviated side; *p* = 0.063). Similarly, in our study, the angle between the lateral lamella and the cribriform lamella was not associated with septal deviation ( *p* = 0.059 for RCPDEG and *p* = 0.064 for LCPDEG). This suggests that the angle between the cribriform lamella and the lateral lamella is less affected by anatomical variations. As a parameter less affected by variations, this area may be considered a keystone especially when working around the anterior ethmoidal artery and the frontal sinus in endoscopic sinus and skull base surgery and may allow for a clear-cut surgical planning.

The present study sought to determine a parameter that changes with the direction of the septal deviation. It was observed that the presence of septal deviation did not change olfactory fossa depths, Keros degrees and lateral lamella - cribriform lamella angle in an independent manner ( *p* > 0.05). However, on the deviated side of the bony septum, olfactory fossa depths and Keros grades changed significantly ( *p* < 0.05). This finding is important to prevent complications when an endoscopic sinus and skull base surgery is performed on the deviated side. Increased length of the lateral lamella at the deviated side has been previously shown by Asal et al. in a CT study of 300 patients 15 and in the study by Saylısoy et al. 14 Other studies that focused on the direction of nasal septum deviation includes one study by Bayrak et al., 16 who did not find a significant association between olfactory fossa depth and the direction and angle of septal deviation on CT scans of 225 patients. On the other hand, in the aforementioned study by Saylısoy et al., it was reported that the ethmoid roof was deeper on the contralateral side of septal deviation. 14 However, our study is more comprehensive because of the higher number of CT images evaluated and of the inclusion of a group without nasal septum deviation. These results show that OF depth and Keros degree increase on the deviated side of the bony part of septum with no significant changes in these parameters on the contralateral side. In a study by Abdullah et al., the angle between the lateral lamella and the cribriform lamella was not significantly associated with Keros degrees, which is consistent with our findings. 17 Anatomical variations including a longer and thinner course of the lateral lamella and a deeper location of the OF may increase the risk of intracranial complications during functional endoscopic sinus surgery (FESS). 18 Thus, these variations should be considered by surgeons prior to endoscopic sinus surgery and endoscopic skull base surgery, especially when operating on the deviated side of the septum.

## Conclusion

Prior to endoscopic sinus surgery and endoscopic skull base surgery, it is extremely important to perform a CT assessment and to determine anatomical structures and variations to prevent complications. In the current study, it was observed that the olfactory fossa depth on the deviated side increased with increased degree of bony septal deviation. This piece of information may be useful for surgeons to avoid complications such as CSF fistula. Although the number of patients included in our study was sufficient to draw some conclusions, further studies with a larger sample size are warranted to evaluate parameters in the sagittal plane as well as in the coronal and axial plane and to investigate septal deviation in combination with other paranasal sinus and skull base variations more extensively. Nevertheless, the lack of any effects of many variables on the angle between the lateral lamella and cribriform lamella suggests that this angle may be used as an important parameter for the CT assessment in endoscopic sinus and skull base surgery.

## References

[1] Beeson W H The nasal septum Otolaryngol Clin North Am 1987 20 04 743 767 3320866

[2] Hatipoğlu H G Cetin M A Yüksel E Concha bullosa types: their relationship with sinusitis, ostiomeatal and frontal recess disease Diagn Interv Radiol 2005 11 03 145 149 16206055

[3] Endoskopik Sinüs Cerrahisi MÖ Baskı. 1999 2 1 17

[4] Mafee M F Preoperative imaging anatomy of nasal-ethmoid complex for functional endoscopic sinus surgery Radiol Clin North Am 1993 31 01 1 20 8419967

[5] Vemuri N V Karanam L SP Manchikanti V Dandamudi S Puvvada S K Vemuri V K Imaging review of cerebrospinal fluid leaks Indian J Radiol Imaging 2017 27 04 441 446 29379240 10.4103/ijri.IJRI_380_16 PMC5761172

[6] Terrier F Weber W Ruefenacht D Porcellini B Anatomy of the ethmoid: CT, endoscopic, and macroscopic AJR Am J Roentgenol 1985 144 03 493 500 3871558 10.2214/ajr.144.3.493

[7] Yeğin Y Çelik M Erdem İ Altıntaş A Şimşek B M Olgun B Nazal septum deviasyon tiplerinin görülme sıklığı 2017

[8] May M Mester S J O'Daniel T G Curtin H D Decreasing the risks of endonasal endoscopic sinus surgery by imaging techniques Oper Tech Otolaryngol–Head Neck Surg 1990 1 02 89 91

[9] Arikan O K Unal B Kazkayasi M Koc C The analysis of anterior skull base from two different perspectives: coronal and reconstructed sagittal computed tomography Rhinology 2005 43 02 115 120 16008066

[10] Hosemann W Draf C Danger points, complications and medicolegal aspects in endoscopik sinus surgery. CMS Curr Top Otorhinolaryngol Head And Neck Surg 2013 12 10.3205/cto000098 PMC3884541 24403974

[11] Skorek A Tretiakow D Szmuda T Przewozny T Is the Keros classification alone enough to identify patients with the ‘dangerous ethmoid’? An anatomical study Acta Otolaryngol 2017Feb;137 02 196 201 27608833 10.1080/00016489.2016.1225316

[12] Krmpotic-Nemanic J Vinter I Judas M Transformation of the shape of the ethmoid bone during the course of life Eur Arch Otorhinolaryngol 1997 254 347 349 9298671 10.1007/BF02630727

[13] Erdem G Erdem T Miman M C Ozturan O A radiological anatomic study of the cribriform plate compared with constant structures Rhinology 2004 42 04 225 229 15626256

[14] Saylisoy S Acar M San T Karabag A Bayar Muluk N Cingi C Is there a relationship between cribriform plate dimensions and septal deviation angle? Eur Arch Otorhinolaryngol 2014 271 05 1067 1071 23982666 10.1007/s00405-013-2661-3

[15] Asal N Bayar Muluk N Inal M Şahan M H Doğan A Arikan O K Olfactory Fossa and New Angle Measurements: Lateral Lamella-Cribriform Plate Angle J Craniofac Surg 2019 30 06 1911 1914 31343591 10.1097/SCS.0000000000005848

[16] Bayrak S Aktuna Belgin C Orhan K Evaluation of the Relationship Between Olfactory Fossa Measurements and Nasal Septum Deviation for Endoscopic Sinus Surgery J Craniofac Surg 2020 31 03 801 803 31934966 10.1097/SCS.0000000000006168

[17] Abdullah B Chew S C Aziz M E A new radiological classification for the risk assessment of anterior skull base injury in endoscopic sinus surgery Sci Rep 2020 10 01 4600 32165705 10.1038/s41598-020-61610-1 PMC7067776

[18] Babu A C Nair M RPB Kuriakose A M Olfactory fossa depth: CT analysis of 1200 patients Indian J Radiol Imaging 2018 28 04 395 400 30662198 10.4103/ijri.IJRI_119_18 PMC6319094

